# Comment on “Innovative logic ‘AND’ gate gene circuits for bladder cancer treatment: preclinical study”

**DOI:** 10.1097/JS9.0000000000003080

**Published:** 2025-08-06

**Authors:** Xingxing Zhang, Helin Zhang, Panfeng Shang

**Affiliations:** a Department of Urology, Fuzhou University Affiliated Provincial Hospital; School of Medicine, Fuzhou University, Fuzhou, China; b Department of Urology, Gansu Province Clinical Research Center for Urinary System Disease, Lanzhou University Second Hospital, Lanzhou, Gansu, China

*Dear Editor,*

We recently read with great interest the article entitled “Innovative logic ‘AND’ gate gene circuits for bladder cancer treatment: preclinical study” by Xu *et al* in the International Journal of Surgery^[1]^. Most conventional molecular-targeted therapies inhibit cancer progression by focusing on single gene targets. However, signaling molecules do not act alone but form networks through interactions during cancer development. To overcome these limitations, the study proposes a synthetic gene circuit, the Logic “AND” Gate Dual-Target Gene Circuit (LAG-DTGC), which integrates multiple signals to achieve comprehensive reprogramming of various signaling pathways in bladder cancer (BLCA) cells. The LAG-DTGC proposed in this study represents a promising new paradigm in cancer therapy, which has the potential to establish a new standard for integrated molecular interventions in BLCA therapy. However, after scrutinizing the manuscript, we identified several points for further improvement. This study is compliant with the Transparency In The reporting of Artificial INtelligence guideline^[2]^.

First, the authors constructed the risk score and Nomogram related to BLCA development by analyzing the TCGA-BLCA dataset and validated them using Cox regression analysis, receiver operating characteristic curves, and calibration curves. The results suggest that the risk score is an independent prognostic factor for BLCA and that the associated Nomogram has good predictive power for overall survival. In fact, this section has little to do with the main theme of this study, and even taking it out would not affect the conclusions of this study. However, if they are retained, we recommend the addition of external datasets to validate the above results for the risk score or compare them with the predictive power of existing models.

Second, by analyzing the gene expression levels of 18 genes in the TCGA database (Fig. 1I), the authors found that most of the genes had abnormal expression patterns in BLCA tissues compared to normal tissues. However, Figure 1I, a heatmap comparing gene expression between the high- and low-risk groups, does not prove that most genes are aberrantly expressed in BLCA. Therefore, this may require additional analytical clarification. To test the specificity of the identified potential 18 BLCA targets, the authors selected two targets (CNTN1 and COL14A1) for validation. Importantly, the LAG-DTGC in the present study was designed based on the selection of CNTN1 and COL14A1 as targets. However, the authors did not mention the rationale for selecting CNTN1 and COL14A1 as targets from the 18 genes. Similarly, the enrichment results for CNTN1 and COL14A1 included a variety of signaling pathways, but the authors did not state the reasons for choosing the transforming growth factor-β/Smad pathway and the mitogen-activated protein kinases/extracellular regulated protein kinases pathway as the pathways targeted for study. Therefore, we suggest giving sound reasons for the above choices to enhance the persuasiveness and credibility of the study.

In addition, there are some problems with the presentation of the figures in the article. For example, the focal magnified area of the four immunohistochemistry (IHC)-stained images in Figure 3I is not bladder epithelial tissue; the IHC-stained images in Figure 3I include images of two non-muscular invasive bladder cancer tissues, whether it would be more pertinent to replace one of them with a representative image of muscular invasive bladder cancer tissue; in Figure 6A–D, the authors divided the treated cells into negative control and BAX groups, respectively, and we believe that it is not appropriate to label the treated group as BAX, and whether it is more appropriate to label it as Genetic Circuit. In addition, we found that the legends of Figures 6A and B and 6C and D are labeled backward. In short, addressing the above issues will improve the accuracy, professionalism, and trust of the readers of the article.

Finally, after infecting the target cells by the lentiviral and adeno-associated virus methods, the authors did not perform a relevant validation of the transfection efficiency, which will probably influence the conclusions of the next phenotyping experiments and animal experiments. Similarly, many of the phenotyping experiments and animal experiments in the study lacked validation at the protein level and transcriptome level. Therefore, we suggest further validation of the above results by using relevant experimental methods to increase the persuasiveness and credibility of the results, such as Western blot and polymerase chain reaction.

In conclusion, LAG-DTGC is a promising new paradigm in cancer therapy. We would like to sincerely thank Xu and colleagues for their joint efforts in taking an important step forward in LAG-DTGC precision therapy for BLCA. However, as the authors stated, while these preclinical results are promising, there are a number of challenges to translating this approach to human patients. For example, the expression levels of genes and signaling networks vary in different cellular lineages in the human body, which may affect the specificity and efficiency of LAG-DTGC. Therefore, more research is needed in the future to optimize the stability and targeted delivery of LAG-DTGC to minimize side effects and improve efficacy against human cancers.

## Data Availability

Not applicable.
